# The progress of tumor vaccines clinical trials in non-small cell lung cancer

**DOI:** 10.1007/s12094-024-03678-z

**Published:** 2024-08-23

**Authors:** Xiaomu Wang, Yunping Niu, Fang Bian

**Affiliations:** 1 Department of Pharmacy, Xiangyang Key Laboratory of Special Preparation of Vitiligo, Xiangyang Central Hospital, Affiliated Hospital of Hubei University of Arts and Science, Xiangyang, Hubei China; 2 Department of Laboratory Medicine, The First People’s Hospital of Xiangyang, Xiangyang, Hubei China

**Keywords:** Tumor vaccines, Clinical trials, Non-small cell lung cancer (NSCLC), Immunotherapy, Studies

## Abstract

**Background:**

Non-small cell lung cancer (NSCLC) remains a significant global health challenge, with high mortality rates and limited treatment options. Tumor vaccines have emerged as a potential therapeutic approach, aiming to stimulate the immune system to specifically target tumor cells.

**Methods:**

This study screened 283 clinical trials registered on ClinicalTrials.gov through July 31, 2023. After excluding data that did not meet the inclusion criteria, a total of 108 trials were assessed. Data on registered number, study title, study status, vaccine types, study results, conditions, interventions, outcome measures, sponsor, collaborators, drug target, phases, enrollment, start date, completion date and locations were extracted and analyzed.

**Results:**

The number of vaccines clinical trials for NSCLC has continued to increase in recent years, the majority of which were conducted in the United States. Most of the clinical trials were at stages ranging from Phase I to Phase II. Peptide-based vaccines accounted for the largest proportion. Others include tumor cell vaccines, DNA/RNA vaccines, viral vector vaccines, and DC vaccines. Several promising tumor vaccine candidates have shown encouraging results in early-phase clinical trials. However, challenges such as heterogeneity of tumor antigens and immune escape mechanisms still need to be addressed.

**Conclusion:**

Tumor vaccines represent a promising avenue in the treatment of NSCLC. Ongoing clinical trials are crucial for optimizing vaccine strategies and identifying the most effective combinations. Further research is needed to overcome existing limitations and translate these promising findings into clinical practice, offering new hope for NSCLC patients.

## Introduction

Lung cancer has emerged as the primary cause of cancer-related fatalities worldwide, accounting for 1.6 million deaths annually [1]. Non-small cell lung cancer (NSCLC) constitutes approximately 85% of all lung cancer cases and is categorized into three types: lung adenocarcinoma, squamous cell carcinoma and large cell lung cancer [2]. According to the latest statistics, the majority of patients are diagnosed at advanced stages, resulting in a mere 5-year survival rate of 15% [3]. NSCLC has diverse molecular subtypes and a complex tumor microenvironment [4]. Current treatment modalities, including surgery, chemotherapy and radiotherapy, often have limited efficacy, and are associated with significant side effects [5]. The immune system plays a crucial role in tumor surveillance and control, but in NSCLC, the immune response is often suppressed or evaded by the tumor. Some molecular-targeted therapies may not provide long-term survival benefits for patients because of drug resistance and the inability of targeted drugs to benefit patients with negative driver gene [6]. Hence, there is a pressing need for an effective method to cure or control NSCLC.

Vaccines have emerged as a promising area of research in the battle against NSCLC, offering the potential to stimulate the immune system to specifically target tumor cells and provide long-lasting protection [7]. Tumor vaccines typically contain tumor-associated antigens (TAAs) or tumor-specific antigens (TSAs). Upon introduction into the body, antigen-presenting cells (such as dendritic cells) uptake these antigens and then process them to form small fragments of antigenic peptides. These antigenic peptide fragments bind to major histocompatibility complex (MHC-I) molecules within the cell, forming MHC-I-antigenic peptide complexes. Subsequently, the MHC-I-antigen-peptide complex is transported to the surface of the antigen-presenting cells. The CD8 + T cell receptors can recognize and bind to the MHC-I-antigenic peptide complex, thereby activating the CD8 + T cells. This activation allows them to proliferate and differentiate into effector CD8 + T cells and CD8 + T cells, which recognize and kill tumor cells [8]. This approach holds the potential to overcome the limitations of conventional treatments and offer a more targeted and personalized therapy.

Currently, based on differences in active ingredients, tumor vaccines are divided into: proteins or synthetic peptides of cancer antigens, cell-based delivery of tumor antigens, and DNA/RNA coding for cancer antigens [9]. Vaccines targeting cancer neoantigens have been considered a new way to direct and enhance immune responses against tumors [10]. Unlike traditional TAAs, neoantigens have stronger immunogenicity and higher affinity for MHC, and are not affected by central immunological tolerance [11]. Over the past 40 years, tumor vaccines across diverse malignant tumors have undergone scrutiny in both preclinical and clinical trials [12]. Numerous diverse vaccine platforms have undergone evaluation in phase II and/or phase III clinical trials. However, only a handful of agents have gained approval from the FDA, and several negative phase III studies have resulted in product discontinuation [13]. Therefore, the primary emphasis in the development of vaccines has been on antigen selection, vaccine design, and combination medication strategies with other drugs (including immunotherapy, chemotherapy, and radiation therapy).

In light of the potential transformative impact of recent advances on the track record of tumor vaccines in NSCLC, especially regarding clinical trials and recent progress of new vaccines, we conducted an analysis of registered clinical trials from Clinical Trials.gov to spotlight the current state of tumor vaccines in NSCLC, aiming to establish a data foundation and information reference based on intelligence for the research and development of anti-tumor drugs.

## Materials and methods

We searched the ClinicalTrials.gov (https://clinicaltrials.gov) website using the keyword “Lung cancer” in the “condition or disease” field and the keyword “vaccine” in the “Other terms” field, restricting the study start time to before July 31, 2023. All registered clinical trials were estimated to obtain records of all research data.

The following clinical trial inclusion criteria were used: (1) a clinical trial must be designed for vaccines; (2) trials involving patients with NSCLC were deemed eligible; and (3) all trials were started before July 31, 2023. The exclusion criteria utilized in the trials were as follows: (1) observational studies; (2) indications included cancers other than NSCLC, excluding solid tumor research; (3) preventive vaccines were not therapeutic vaccines; and (4) laboratory analysis. The following information and data were collected for analysis: registered number, study title, study status, vaccine types, study results, conditions, interventions, outcome measures, sponsor, collaborators, drug target, phases, enrollment, start- date, completion date and locations. Microsoft Office Excel 2010 was used for data processing and analysis. Frequencies and percentages were reported for categorical variables based on descriptive analyses.

**Fig. 1:**
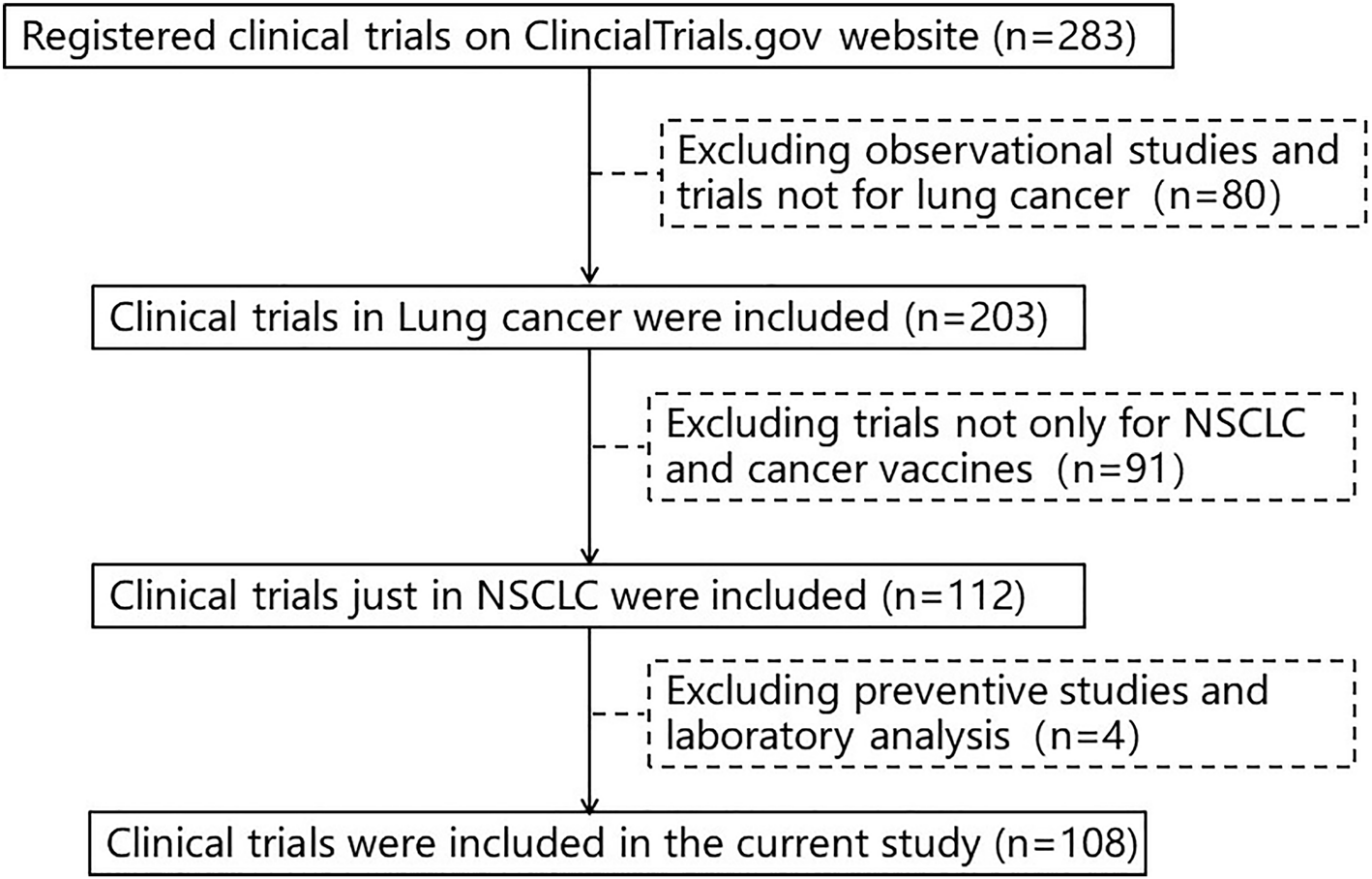
Data-retrieval process

We screened 283 clinical trials registered on ClinicalTrials.gov through July 31, 2023. After excluding 39 observational trials, 41 trials not for lung cancer and 84 trials not specifically aimed at NSCLC, 158 clinical trials in NSCLC were included. After further investigation, seven trials not for vaccines, two trials for laboratory analysis and two trials for cancer prevention were excluded. Ultimately, a total of 108 registered trials were accessed (Fig. 1). This study involved only publicly available data from ClinicalTrials.gov, and did not reflect subject's personal information. Therefore, this study was exempt from institutional review board review.

### Review to literature

This section discusses the clinical research literature on NSCLC vaccines, classifies the types of vaccines and summarizes the results of the relevant clinical studies. Details are shown in Table 1.

**Table 1:** Current analysis of relevant papers

References	Paper	Classification	Results	Identifier
[16]	Telomerase peptide vaccination: a phase I/II study in patients with non-small cell lung cancer	Peptide/protein vaccines	GV1001 was immunogenic and safe	/
[17]	Long-Term Outcomes of a Phase I Study With UV1, a Second Generation Telomerase Based Vaccine, in Patients With Advanced Non-Small Cell Lung Cancer	Peptide/protein vaccines	Treatment with UV1 was well tolerated with no serious adverse events observed	NCT01789099
[19]	Randomized open-label controlled study of cancer vaccine OSE2101 versus chemotherapy in HLA-A2-positive patients with advanced non-small-cell lung cancer with resistance to immunotherapy: ATALANTE-1	Peptide/protein vaccines	OSE2101 increased survival with better safety compared to CT	3(chemotherapy)
[22]	Updated survival analysis in patients with stage IIIB or IV non-small-cell lung cancer receiving BLP25 liposome vaccine (L-BLP25): phase IIB randomized, multicenter, open-label trial	Peptide/protein vaccines	L-BLP25 plus BSC significantly prolonged median survival time compared with BSC alone	/
[23]	Tecemotide (L-BLP25) versus placebo after chemoradiotherapy for stage III non-small-cell lung cancer (START): a randomised, double-blind, phase 3 trial	Peptide/protein vaccines	No significant difference was found in overall survival with the administration of L-BLP25 after chemoradiotherapy compared with placebo	NCT00409188
[26]	A Phase III Clinical Trial of the Epidermal Growth Factor Vaccine CIMAvax-EGF as Switch Maintenance Therapy in Advanced Non-Small Cell Lung Cancer Patients	Peptide/protein vaccines	Switch maintenance with CIMAvax-EGF was well tolerated	/
[27]	Safety and effectiveness of CIMAvax-EGF administered in community polyclinics	Peptide/protein vaccines	CIMAvax-EGF was also proved to be safe	/
[31]	Pilot study of 1650-G: a simplified cellular vaccine for lung cancer	Tumor cell vaccines	1650-G was safe and generated a robust and unequivocal immunological response in 6/11 of immunized patients	
[33]	Viagenpumatucel-L (HS-110) plus nivolumab in patients with advanced non-small cell lung cancer (NSCLC) after checkpoint inhibitor treatment failure	Tumor cell vaccines	Good patient tolerance and safety characteristics	/
[34]	A phase III study of belagenpumatucel-L, an allogeneic tumour cell vaccine, as maintenance therapy for non-small cell lung cancer	Tumor cell vaccines	Belagenpumatucel-L could significantly increase in OS	/
[35]	A phase I/randomized phase II study of GM.CD40L vaccine in combination with CCL21 in patients with advanced lung adenocarcinoma	Tumor cell vaccines	No significant association was found between vaccine immunogenicity and outcomes	NCT01433172
[37]	A pilot study of an autologous tumor-derived autophagosome vaccine with docetaxel in patients with stage IV non-small cell lung cancer	Tumor cell vaccines	DRibble vaccine given with GM-CSF appeared safe and capable of inducing an immune response against tumor cells in this small, pilot study	NCT00850785
[41]	Phase I Trial of sequential administration of recombinant DNA and adenovirus expressing L523S protein in early stage non-small-cell lung cancer	DNA vaccines	the administration of pVAX/L523S and Ad L523S as a combined vaccine was well-tolerated and that no notable toxicity had been observed	/
[47]	Phase Ib evaluation of a self-adjuvanted protamine formulated mRNA-based active cancer immunotherapy, BI1361849 (CV9202), combined with local radiation treatment in patients with stage IV non-small cell lung cancer	RNA vaccines	Good patient tolerance	NCT01915524
[53]	Phase I Trial of Intratumoral Injection of CCL21 Gene-Modified Dendritic Cells in Lung Cancer Elicits Tumor-Specific Immune Responses and CD8( +) T-cell Infiltration	Viral vector vaccines	Injection increased infiltration of CD8 + T cells and elevated expression of PD-L1 mRNA	NCT01574222
[56]	Personalized neoantigen pulsed dendritic cell vaccine for advanced lung cancer	DC vaccines	Neo-DCVac was feasible, safe, and capable of eliciting specific T-cell immunity and therapeutic benefit	NCT02956551

## Results

###  Basic characteristics

**Fig. 2:**
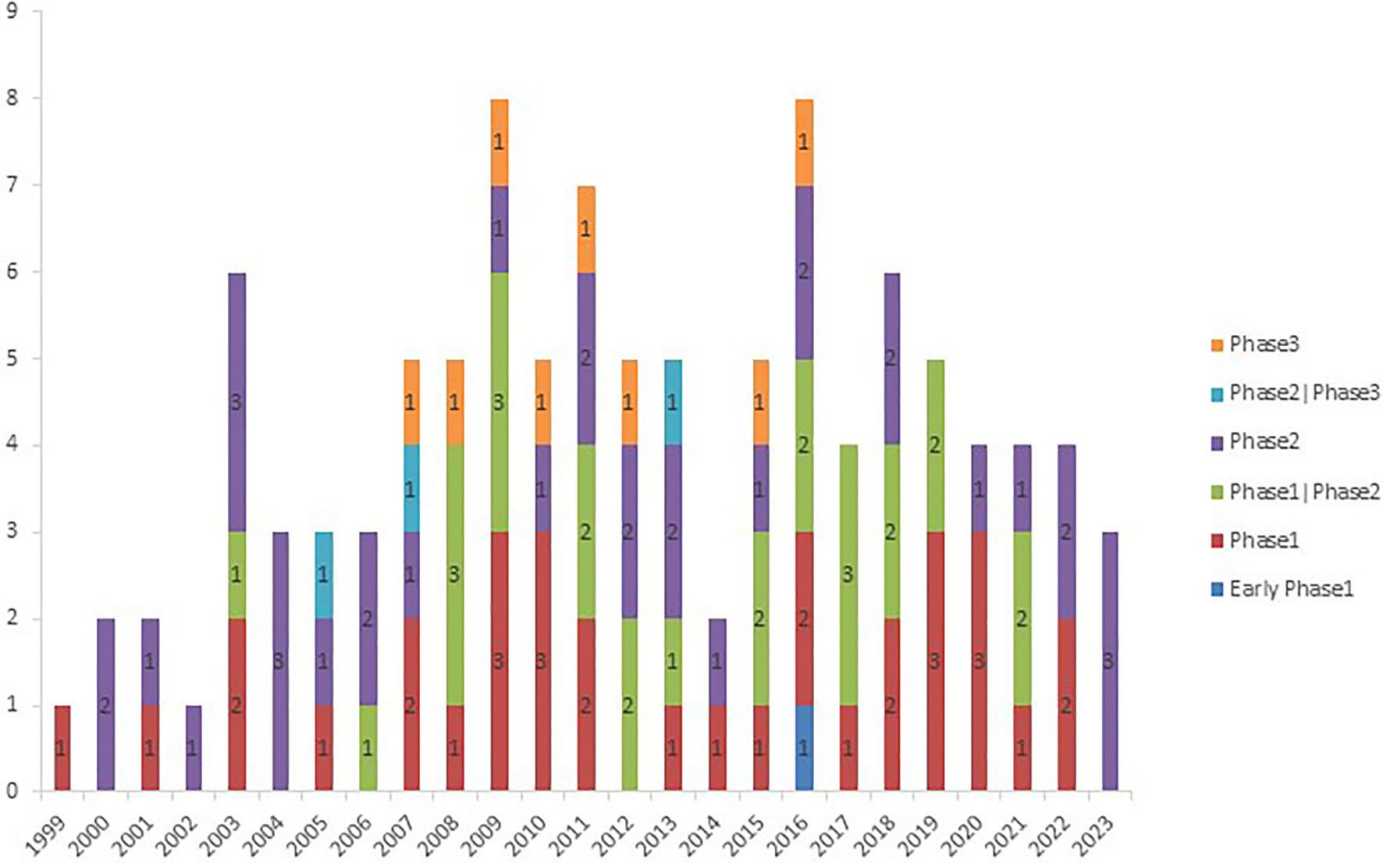
Number of registered clinical trials grouped by study phase

Of the 108 clinical trials, 32 trials (29.6%) were started from 1999 to 2008, 56 trials (51.9%) between 2009 and 2018, and 20 trials (29.06%) until July of 2023. Besides, merely 1 trial (0.9%) was in the early phase 1. Thirty-three (30.6%), 26 (24.1%), and 35 (32.4%) trials were in phase 1, phase 1/2, and phase 2, respectively. Three (2.8%) and 8 (7.4%) trials were phase 2/3 and phase 3 trials (detailed data are shown in Fig. 2). Most NSCLC clinical trials were primarily conducted in the United States (50, 46.3%), followed by Japan (9, 8.3%) and China (8, 7.4%).

All the clinical trial types were interventional trials, the majority of which were single group (53, 49.1%), followed by parallel group (31, 28.7%) and sequential group (6, 5.6%). The primary purpose of the majority of trials (105, 97.2%) involved providing treatment. Only 1 trial (0.9%) was prevention. Except the allocation method of 53 trials were not clear, 35 trials (32.4%) were randomized, and 20 (18.5%) were non-randomized. Among these studies, one single (0.9%), two double (1.8%), two triple (1.8%) and seven quadruple (6.5%) trials were masking, others were without masking or unknown. Moreover, the majority of vaccine clinical trials (92, 85.2%) had no results, and only 16 trials (14.8%) uploaded results.

**Fig. 3:**
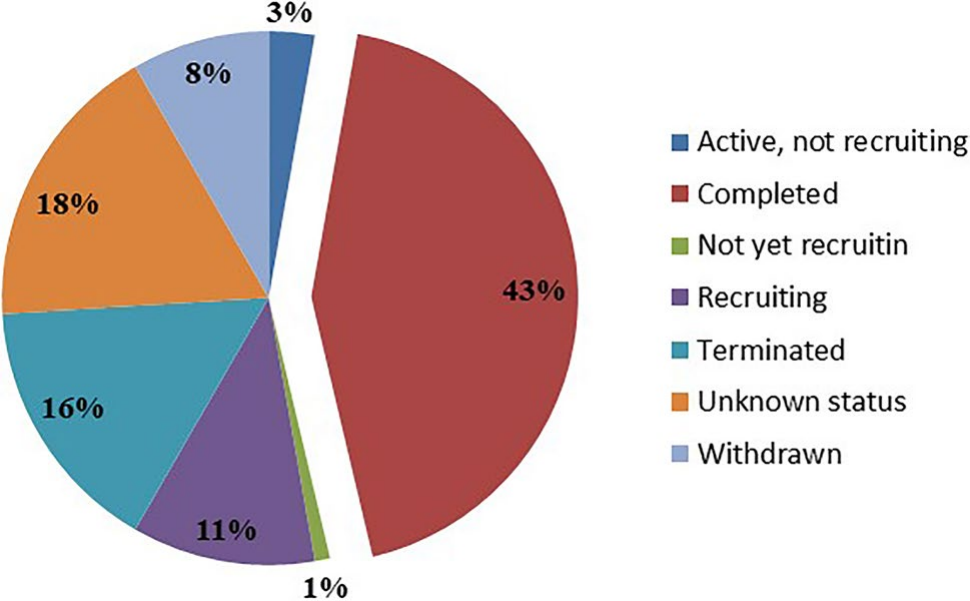
The proportion of registered clinical trials grouped by status

As shown in Fig. 3, approximately half of the vaccine clinical trials (47, 43.5%) were complete; 12 trials (11.1%) were still in the recruiting stage; 17 trials (15.7%) were terminated; 9 trials (8.3%) were withdrawn; 4 trials (3.7%) were ongoing, but participants had not been recruited; 1 trial had not yet recruited. Over 50% of the trials (61, 56.5%) recruited fewer than 60 individuals, 23 trials (21.3%) recruited 61–200 participants, and 8 trials (7.4%) recruited more than 200 individuals.

###  Vaccine types of included trials

**Fig. 4:**
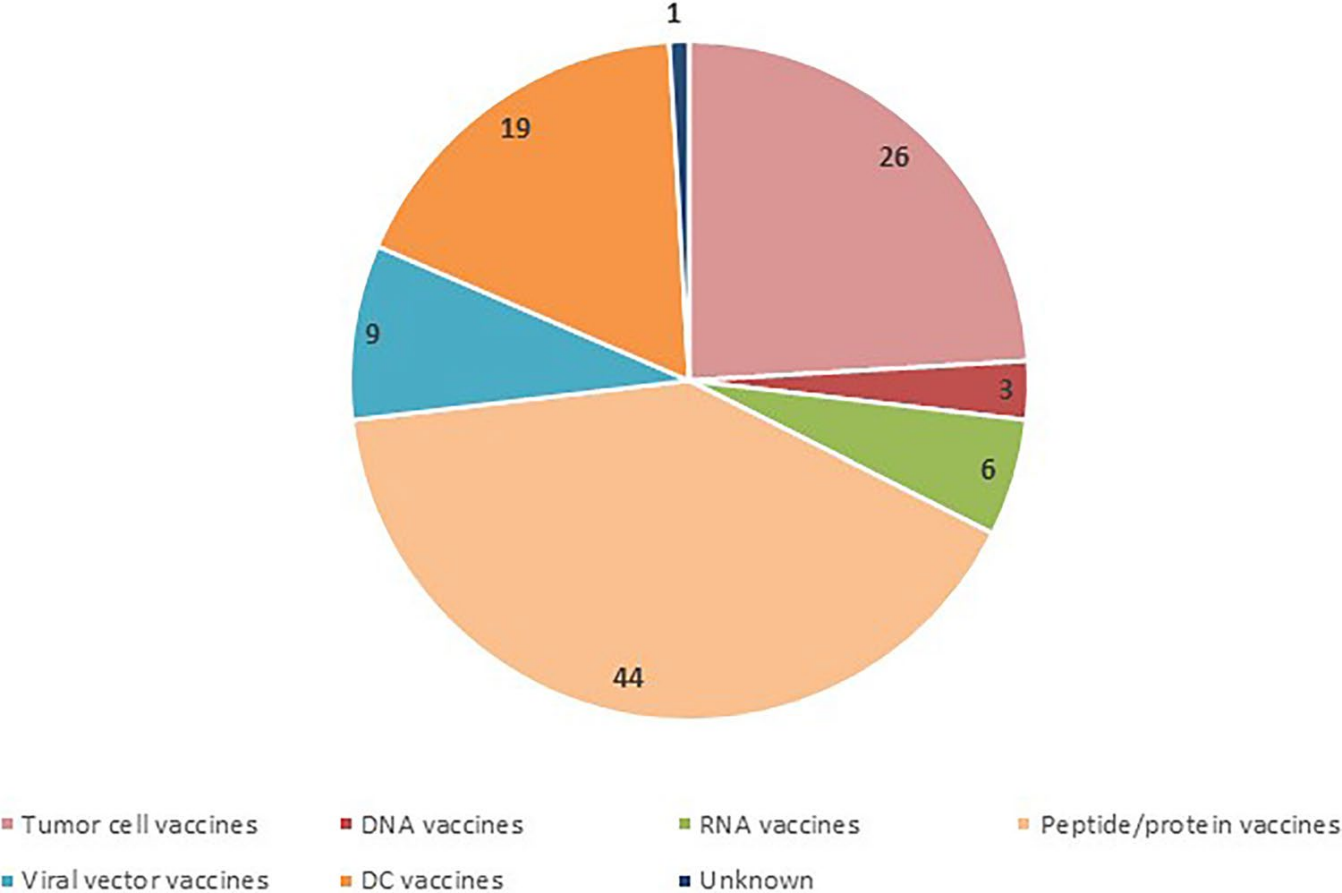
The number of various types of therapeutic vaccines

The identification of antigens and vaccine vectors is pivotal for the design and development of tumor vaccines [14]. The following six different types of tumor vaccines have been designed that vary depending on the form of the delivered antigen: tumor cell vaccines (26, 24.1%), DNA vaccines (3, 2.8%), RNA vaccines (6, 5.6%), peptide/protein vaccines (44, 40.7%), viral vector vaccines (9, 8.3%) and dendritic cell (DC) vaccines (19, 17.6%). Among these clinical trials, peptide/protein vaccines account for the largest proportion, followed by tumor cell vaccines and DC vaccines. Detailed data are shown in Fig. 4. Each type of vaccine is described in detail as below.

###  Peptide/protein vaccines

Peptide/protein vaccines can effectively stimulate T cells, leading to an augmented immune response [14]. Synthetic peptides typically consist of 20–30 amino acids and specifically target epitopes related to tumor antigens [15]. In recent years, peptide vaccines have been under development for use as active immunotherapy in the treatment of NSCLC. GV1001 (NCT01579188) is a peptide-based vaccine that corresponds to hTERT and has strong human leukocyte antigen (HLA) class II binding properties. A phase I/II study demonstrated that GV1001 was immunogenic and safe to use in patients with NSCLC [16]. In clinical trials, peptide-based vaccines typically have multiple epitopes for various targets. UV1(NCT01789099), a peptide-based vaccine consists of three synthetic long peptides containing multiple epitopes. In a phase I study of UV1 vaccination with GM-CSF as adjuvant, patients who received UV1 treatment exhibited good tolerance [17]. These findings provide support for further clinical trials involving UV1 vaccination combined with immune checkpoint blockade (NCT05344209). Personalized neoantigen vaccines, as novel peptide-based tumor vaccines, have attracted attention due to its safety, effectiveness and ability to induce stronger immune response [18]. OSE2101 (NCT02654587), is a neoantigen vaccine specifically for NSCLC. A randomized open-label controlled study suggested that OSE2101 significantly increased survival compared to CT in HLA-A2-positive individuals with advanced NSCLC and secondary resistance to immunotherapy [19].

An alternative strategy involves the utilization of whole proteins as carriers for antigens, such as microemulsions, liposomes and various microparticle platforms [20]. L-BLP25 (Tecemotide) is a liposome vaccine containing the BLP25 lipopeptide, three lipids, and the immune adjuvant monophosphate lipid A [21]. A randomized controlled phase 2b study (NCT00157209) investigating the immunotherapeutic potential of L-BLP25 in patients with NSCLC revealed that individuals who received a combination of L-BLP25 and best supportive care experienced a significantly prolonged median survival time compared to those who solely received best supportive care [22]. However, not all results are promising. Another trial of L-BLP25 versus placebo or cyclophosphamide in patients with unresectable stage III NSCLC (NCT00409188) suggested no notable disparity in overall survival rate between the two groups [23]. Giving vaccine therapy together with bevacizumab (NCT00828009) might introduce fresh perspectives on the function of L-BLP25 for NSCLC patients. Another protein vaccine is CIMAvax-EGF (NCT02187367 and NCT01444118), which consists of a carrier protein conjugated with recombinant EGF and emulsified in Montanide ISA5 [24]. CIMAvax-EGF was approved as a maintenance treatment for patients with stage IIIB/IV NSCLC, after front-line chemotherapy [25]. In the Phase III trial, the 5-year survival rate was observed to be 14.4% among vaccinated subjects, compared to 7.9% among controls [26]. CIMAvax-EGF was also proved to be safe in a real-world trial, and the most common adverse events included mild-to-moderate injection site reactions, fever and headache [27]. Furthermore, racotumomab (NCT01240447, NCT01460472), an innovative vaccine consisting of a unique monoclonal antibody that targets NeuGcGM3, has been approved as an effective immunotherapeutic approach for the treatment of advanced NSCLC in Latin America [28]. These findings show that peptide/protein vaccines exhibit potential in offering efficient approaches for the treatment of NSCLC. Details of the peptide/protein vaccine clinical trials in NSCLC are shown in Table 2.

**Table 2:** Details of the peptide/protein vaccine clinical trials in NSCLC

Vaccine/antige	Drug target	Immune response	Identifier	Combination therapy
Tecemotide (L-BLP25)	MUC1	MUC1-specific T cells	NCT00157209, NCT00960115 NCT00157196,NCT00409188 NCT01015443	5(chemotherapy)
MUC1 PolyICLC	MUC1	MUC1-specific T cells	NCT01720836	/
EGF-rP64K	EGF	GARs and anti-EGF antibodies	NCT00516685,NCT01444118 NCT02187367	3(chemotherapy)
GV1001	hTERT	GV1001-specific responses	NCT01579188	/
UV1	hTERT	UV1-specific T cells	NCT05344209,NCT01789099	1( PD-1/PD-L1)
UCPVax	hTERT	UCPVax-specific T cells	NCT04263051,NCT02818426	1 (PD-1 and chemotherapy)
Vx-001	hTERT	Vx-001-specific T cells	NCT01935154	/
Racotumomab	NeuGcGM3	anti-NeuGcGM3 response	NCT01240447,NCT01460472	/
OSE2101	ACE,HER2, MAGE2, MAGE3, P53	Tumor specific T cells	NCT02654587	1(chemotherapy)
IMU-201	PD-1	Blocking PD-1 signal	NCT04432207	1 (PD-L1)
(HLA)-A*0201	URLC10, VEGFR1, VEGFR2	specific CTL response	NCT00673777, NCT01069640, NCT01949701, NCT03970746	1 (PD-L1)
(HLA)-A*2402	URLC10, CDCA1, KIF20A, KOC1, TTK, VEGFR1, VEGFR2	specific CTL response	NCT01950156, NCT01069575, NCT00674258, NCT00874588 NCT00633724, NCT01592617	/
EP-2101	10 Targeted Peptides	EP-2101-specific T cells	NCT00104780, NCT00054899	/
NEO-PV-01	20 Targeted Peptides	NEO-PV-01-specific T cells	NCT03380871	1(PD-1 and chemotherapy)
neoantigen	EGF	anti-EGF antibodies	NCT04487093, NCT04397926	2 (EGFR-TKI)
Cyclin B1 Peptide	Cyclin B1	specific CTL response	NCT01398124	/
KRAS	KRAS	anti-KRAS antibodies	NCT05254184, NCT00005630	1(PD-1/PD-L1 and CTLA-4) 1(chemotherapy)
antibody 11D10	/	specific CTL response	NCT00006470	1(radiation)
FRAME-001	/	specific CTL response	NCT04998474	1 (PD-L1)
Gsk2302032A	PRAME	specific CTL response	NCT01853878, NCT01159964	/
GSK249553	/	specific CTL response	NCT00290355	/
TEIPP24	/	specific CTL response	NCT05898763	/

### Tumor cell vaccines

Tumor cell vaccines are primarily derived from autologous or allogeneic tumor cells [29]. Among the 26 registered cell vaccine clinical trials, the majority involved allogeneic tumor cells. Allogeneic cellular vaccines utilize cancer cells from different individuals as the antigen source. These cancer cells, obtained from one patient, undergo necessary modifications and processing before being administered to another patient with the same type of cancer [30].

The1650-G vaccine (NCT00654030), is an allogeneic cellular vaccine using granulocyte macrophage colony stimulating factor as an adjuvant. It has been demonstrated to be safe and capable of eliciting a robust and unequivocal immunological response in 6 out of 11 subjects [31]. Based on these promising results, more combination therapies, including the combination with an oral medication known as beta glucan (NCT01829373), are being used to improve immune recognition ability. Similarly, Viagenpumatucel-L (HS-110) is an allogeneic cellular vaccine obtained by transfecting the DNA of the gp96-Ig fusion protein into human lung adenocarcinoma (AD) cell lines [32]. DURGA (NCT02439450) assessed the safety and effectiveness of Viagenpumatucel-L combined with navumab in the treatment of advanced lung adenocarcinoma after treatment, showing the good patient tolerance and safety characteristics [33]. Belagenpumatucel-L (Lucanix), another genetically modified allogeneic cellular vaccine, could significantly increase in OS among NSCLC patients after the end of first-line chemotherapy [34]. These findings suggest that allogeneic cellular vaccines have a promising therapeutic future against NSCLC. In addition, GM.CD40L has been focused on generating an allogeneic tumor cell-based vaccine formulation. However, there are no clear results yet. One study compared GM.CD40L versus GM.CD40L plus CCL21 in lung adenocarcinoma patients who received with ≥ 1 line of treatment (NCT01433172). The results did not reveal any significant correlations between vaccine immunogenicity and outcomes based on limited biopsy samples [35]. Additional research has been conducted, such as lung adenocarcinoma cells that have been transfected with hCD40L and hGM-CSF (NCT00601796) or B7.1 (CD80) and HLA A1 (NCT00534209).

Autologous cellular vaccines originate from the patient's own tumor tissue or cells [36]. A Dribble vaccine (NCT00850785), derived from tumor cells obtained from pleural effusions, was administered to patients with advanced NSCLC to evaluate its safety and immune response. Although all four patients showed signs of particular antibody responses against potential tumor antigens, a small population could not prove the effectiveness of the vaccine [37]. Further studies on the combination of Dribble with DC-CIK (NCT03057340) or HPV vaccines (NCT01909752) with a larger sample size have been conducted. In addition, the HSPPC-96 vaccine is a gp96 protein derived from autologous tumor tissue carrying antigenic peptides [38]. A phase II study enrolling NSCLC patients was performed to assess overall survival in individuals receiving HSPPC-96 (NCT00098085). More autologous cellular vaccines were used to conduct feasibility studies on NSCLC patients, such as the GVAX vaccine (NCT00074295), the DNP-modified NSCLC vaccine (NCT00298298) and the CG8123 vaccine (NCT00089726).

### DNA vaccines

DNA vaccines can insert exogenous antigen genes into plasmids, and then introduce the plasmids into vivo to express antigen proteins in host cells, inducing immune responses [39]. For example, semi-allogeneic human fibroblasts (MRC-5) was transfected with DNA (NCT00793208). The application of molecular recombinant DNA technology enables the flexible design of DNA vectors for encoding a diverse range of antigens and immunomodulatory molecules [40]. The lung cancer antigen L523S was initially identified by screening genes that are differentially expressed in cancer tissue compared to normal tissue. A phase I clinical trial (NCT00062907) was conducted to assess the safety and immunological reactions after administering recombinant DNA and adenovirus containing the L523S protein in individuals diagnosed with early stage NSCLC. The finding suggested that the administration of pVAX/L523S and Ad L523S as a combined vaccine was well-tolerated and that no notable toxicity had been observed [41]. Including the antigens, the vector has the ability to incorporate diverse immunomodulatory molecules such as GM-CSF [42]. GM-CSF can enhance the effect of DNA vaccines on immune responses [43]. STEMVAC (NCT05242965), a multiple antigen DNA vaccine, containing five proteins (CDH3, SOX2, YB-1, MDM2 and CD105), was injected into NSCLC patients along with GM-CSF.

### RNA vaccines

RNA vaccines have attracted considerable attention in the past few years. They are mainly divided into three types: non-replicating unmodified mRNA, modified mRNA and self-amplifying mRNA derived from viruses [44]. By targeting TAAs or personalized neoantigens, RNA vaccines can initiate specific immune response against tumor [45]. RNA vaccines against TAAs offer new possibilities for personalized therapy. CV9202 is an RNA mixture encoding six overexpressed antigens in lung cancer cells, including MUC1, NY-ESO-1, MAGE-C2, MAGE-C1, Survivin and 5T_4_ [46]. Researchers investigated the safety and effectiveness of combining CV9202 with localized radiation in patients with NSCLC (NCT01915524). The results revealed that stable disease was achieved as the best overall response in 46.2% of patients, and an impressive 84% of patients exhibited enhanced immune responses specific to antigens [47]. The findings provide support for further exploration of mRNA-based immunotherapy in NSCLC, including its potential combinations with immune checkpoint inhibitors (NCT03164772). Similarly, BNT116 is developed by BioNTech based on its FixVac platform. The combination of BNT116 and the PD-1 inhibitor cemiplimab for the treatment of advanced NSCLC patients (NCT05142189 and NCT05557591), is currently undergoing evaluation. The rapid development of mRNA platforms has made it possible to develop personalized vaccines [48]. BNT122, an individualized neoantigen vaccine based on uridine mRNA-lipoplex nanoparticles, has already been proved to greatly enhance the activity of T cells and substantially prolong the time to relapse in postoperative pancreatic cancer patients [49]. Now, this approach has been used to evaluate the effectiveness and safety when combined with Atezolizumab for NSCLC patients (NCT04267237).

### Viral vector vaccines

The virus vector vaccine can use an attenuated synthetic virus as the carrier of tumor antigen genes, which can be introduced into host cells for expression after modification, thereby stimulating the body to produce anti-tumor immune responses [50]. Now, more and more types of virus vectors are being developed to achieve optimal therapeutic effects. TG4010 is a recombinant modified bovine pox virus suspension capable of encoding MUC1 tumor related antibodies and interleukin-2 (IL-2) [51]. A randomized phase IIb trial showed that adding the TG4010 vaccine could significantly improve patient' progression free survival (PFS) and remission rates [52]. It becomes a highly suitable candidate drug for the first-line treatment of NSCLC in combination with other therapies, including immune checkpoint inhibitors (NCT02823990, NCT03353675) and chemotherapy (NCT00415818). Another viral vector vaccines MG1-MAGEA3 (NCT02879760), based on genetically engineered Maraba MG1 rhabdovirus to carry cancer cell antigen MAGEA3, has been combined with an anti-PD1 monoclonal antibody for NSCLC treatment. Adenoviruses are often used as vectors for the transduction of certain genes. In a phase I clinical trial, sixteen individuals with stage IIIB/IV NSCLC received intratumoral injections of the autologous dendritic cell-adenovirus CCL21 vaccine (NCT01574222), resulting in increased infiltration of CD8 + T cells and elevated expression of PD-L1 mRNA [53]. Other adenovirus vaccines, including the chimpanzee adenovirus oxford 1 (ChAdOx1) vaccine (NCT04908111), the recombinant fowlpox GM-CSF vaccine (NCT00091039), the attenuated measles vaccine (NCT00828022) and the live-attenuated Double-deleted Listeria JNJ-64041757 vaccine (NCT02592967), are also being used to improve NSCLC patients’ immunity.

### DC vaccines

DCs are widely recognized as the most functional specialized APCs in the body and possess the capacity to initiate naive T, CD4 + T, and B cells, thereby initiating the immune response [54]. We identified 19 (17.6%) registered DC vaccine clinical trials in NSCLC. According to the difference between the method of transmitting TAAs and DC molecular modifications, including autologous DC pulsed with TAAs (NCT03371485), TAA-peptide (NCT00019929), autologous cancer cell lysates (NCT00023985, NCT00098917 and NCT00103116), and DC derived exosomes (NCT01159288). Besides, multiple ongoing clinical trials are using novel antigens (NCT03205930, NCT04082182 and NCT04078269). It was reported that antigen-loaded DC vaccines elicited more robust immune responses compared to vaccines consisting of antigens and adjuvants [55]. In a recent Phase I clinical trial (NCT02956551), the safety and efficacy of personalized neoantigen autologous DC vaccine were investigated in the treatment of 12 cases of severe metastatic NSCLC. The results showed that the objective effectiveness rate was 25%, with a disease control rate of 75% [56]. Based on these exciting effects, personalized neoantigen autologous DC vaccines may become a promising therapeutic method in treatment of NSCLC patients. Besides, DC vaccines are usually combined with other therapies to improve their effectiveness and safety. Common ones are chemotherapy drugs, such as cyclophosphamide (NCT05195619 and NCT02419170), carboplatin (NCT02669719) and celecoxib (NCT00442754). DC vaccines can also be used with the standard care (NCT02470468) and CIK cells (NCT02688686). Furthermore, the coadministration of DC vaccines and PD-1/PD-L1 checkpoint inhibition (NCT03546361 and NCT02495636) is expected to augment the immune response.

## Discussion

To date, the treatments for NSCLC include surgical intervention for early-stage disease, as well as chemotherapy and radiation therapy for advanced-stage cancer [57]. Nevertheless, these therapies show only limited improvements in survival. Cancer vaccination represents a therapeutic modality aimed at eliciting immune system activation against malignant cells, thereby offering promising avenues for effective cancer treatment [20]. Currently, numerous ongoing trials are exploring different approaches for developing vaccine treatments. We analyzed 108 tumor vaccines clinical trials in NSCLC from 1999 to 2023; only a handful of these immunotherapies have progressed to phase III trials; most clinical trials do not have results and are still at an exploratory stage. The efficacy of these therapies may be constrained by interindividual variability in the immune system response and the challenges associated with identifying suitable antigens. Hence, many endeavors have been undertaken to enhance the effectiveness of immunotherapy. Various delivery approaches, including tumor cell-based, peptide/protein-based, DNA-based, RNA-based, viral vector-based, and DC-based vaccines, present unique benefits, as discussed below.

Peptide/protein vaccines account for the largest proportion of these therapeutic vaccines. Most peptide vaccines are chemically synthesized [15]. Adjuvants, such as GM-CSF, poly-ILC and AS15, are essential components of cancer vaccines by activating innate immune pathways to enhance immune responses [58]. Alum and Montanide^™^ ISA51 have been used as CIMAvax-EGF adjuvants, which improved the safety and effectiveness of the vaccine in 106 NSCLC patients [59]. Peptide vaccines present numerous benefits, including ease of synthesis, low production costs and high chemical stability [60]. Some preliminary clinical trials, such as L-BLP25 and CIMAvax-EGF, have yielded encouraging results. However, most trials have failed when they reached phase III. For example, a phase I trial was conducted to evaluate the safety and immunogenicity of a combination therapy comprising recombinant PRAME and the immune-boosting agent AS15. Further investigation of the vaccine was terminated due to the absence of comparable outcomes in 60 NSCLC patients [61]. Some challenges limit the development of peptide/protein vaccines. First, peptide/protein vaccines typically target a limited number of TAA epitopes, and are unable to achieve multivalent antigen-specific CTL responses [62]. Second, the binding of peptides to particular HLA molecules for immune recognition restricts their applicability in patients who possess compatible HLA types, potentially excluding a considerable proportion of patients [63]. Third, the delivery methods of the vaccine may cause adverse events due to its potential toxicity. Currently, various administration methods, including the use of transdermal patches, subcutaneous injection and intravenous injection, have been extensively explored to improve vaccination safety and efficacy [64].

Tumor cell vaccines typically employ TAAs derived from isolated tumor cells as antigen sources [30]. Unlike targeting a single protein or peptide tumor antigen, this method enables the display of all potential antigens expressed by cancer cells, eliminating the necessity of identifying the most suitable antigen for a particular type of cancer. This elicits a broader immune response by activating T cells, B cells, and NK cells [65]. The Lucanix^™^ vaccine have been shown promising outcomes, as indicated by a noteworthy 2-year survival rate of 47% among 41 patients with advanced-stage NSCLC who were administered doses equal to or exceeding 2.5 × 10^7^ cells per injection [66]. The preparation of cell vaccines, however, requires substantial resources and expertise, resulting in high production costs. Therefore, it is imperative to improve vaccine preparation by streamlining the production and distribution processes while capitalizing on advancements in personalized medicine [67]. Receiving an injection of GVAX, a vaccine which was developed by combining autologous tumor cells with an allogeneic cell line that secretes GM-CSF, resulted in enhanced survival rates for patients with metastatic NSCLC who had previously experienced chemotherapy failure. The improvement in survival was observed to be dose-dependent, with patients experiencing 471 days of survival compared to 174 days [68].

The utilization of DNA vaccines has emerged as a compelling approach for cancer immunotherapy due to their efficacy, stability, scalability and low cost [69]. The design of plasmid DNA allows it to function as both an antigen and an adjuvant, owing to the presence of unmethylated CpG motifs in the delivered DNA that stimulate immune response pathways [70]. Although DNA vaccines can induce a robust CD8 + T cell response against neoantigens in comparison to peptide or RNA vaccines, they have no ability to completely escape recognition and attack by the immune system [14]. Consequently, few DNA vaccines have progressed beyond phase I or phase II clinical trials, with only three trials involving DNA vaccines in NSCLC. To enhance efficacy, DNA vaccines are frequently combined with other adjuvants, such as cytokines [39]. The DNA vaccine STEMVAC, administered alongside GM-CSF, enhances the immune response in patients with stage IV non-squamous NSCLC. However, concerns exist regarding the potential integration of plasmid DNA into the chromosome, the utilization of genes that encode cytokines, and the potential adverse effects of the antigen [71].

RNA vaccines were first reported in the late 1990s, and subsequently outpaced traditional vaccine platforms owing to their remarkable efficacy, secure delivery methods, accelerated development prospects, and economically viable production processes [44, 72]. Over the past 2 years, SARS‐CoV‐2 mRNA vaccines have made significant contributions against SARS‐CoV-2, leading to a surge in mRNA vaccine research, particularly in the field of cancer vaccines [73]. In contrast to DNA vaccines, mRNA vaccines can undergo translation in both dividing and non-dividing cells, without integration into the genome [74]. The self-adjuvanted mRNA vaccine CV9201, which encodes diverse cancer antigens, has been evaluated in phase I/IIA trials conducted on patients with advanced NSCLC. These trials demonstrated favorable tolerability profiles and elicited antigen-specific cellular and humoral immune responses [75]. However, the application of mRNA vaccines has been limited by instability, inherent immunogenicity and ineffective in vivo delivery [44]. Extensive research has been conducted to address these issues through modifications in mRNA structure, formulation techniques, and administration routes [76]. Besides, the co-administration of RNA vaccines alongside other immunotherapeutic approaches, such as chemotherapy, radiotherapy, checkpoint inhibitors and endocrine therapy, can enhance the immune response and increase the probability of complete tumor elimination [47].

Viral vector vaccines employ genetically modified viruses to directly stimulate the immune response [77]. When these viruses infiltrate cancer cells, they replicate and express TAAs. These TAAs are then presented to immune cells, triggering an inflammatory response and the release of pro-inflammatory cytokines and chemokines [78]. This approach selectively identifies and eliminates cancer cells, providing a highly targeted strategy for cancer immunotherapy [79]. Currently, adenoviruses and cowpox viruses, such as Ad-MAGE3 and TG4010, have been extensively studied as viral vectors due to their remarkable ability to stimulate the activation of CTLs [80]. A placebo-controlled, randomized phase II study reported that TG4010 elicited an expansion of specific immune response and improved outcomes in advanced NSCLC patients [81]. The limitations of viral vector vaccines may depend on pre-existing immunity to the virus and potential adverse effects associated with the viral vector [82]. Thus, various strategies including the combination with other immunotherapies, are being developed to improve the effectiveness of viral vector vaccines.

DC vaccines are prepared by extracting autologous monocytes from a patient’s peripheral blood and then co-culturing them with antigens and adjuvants. These cultured DCs are matured and loaded with TAAs [83]. Subsequently, they are reintroduced into the patient's body where they migrate to the CD8 + and CD4 + T cells to initiate anti-tumor immunity [84]. Neo-DCVac, a personalized neoantigen peptide pulsed autologous DC vaccine, has been demonstrated to be safe and effective in advanced NSCLC patients [56]. While Neo-DCVac has shown success and promising initial results in phase I/II clinical trials, it is challenging to make direct comparisons or draw broad conclusions from these trials due to significant differences in the production methods and ingredients used in DC vaccines. The development of DC vaccines is hindered by the complexity and high cost of vaccine manufacturing, as well as the difference between individual patients [85]. The aforementioned factors have contributed to the necessity for refinement in both the manufacturing and administration processes of DC vaccines, aiming to optimize their efficacy.

### Challenges and future directions

Despite the advancements achieved in the field of NSCLC vaccines, the limitations and potential adverse effects associated with these vaccines continue to present significant challenges. While current vaccines demonstrate efficacy in certain patients, the overall response rate remains relatively modest, and not all NSCLC patients experience significant therapeutic benefits. And substantial differences exist in individual patient responses to the vaccine, impacting its widespread applicability. Moreover, immunosuppressive factors in the tumor microenvironment may impair vaccine-induced immune response. In addition, the high heterogeneity of tumor cells makes it difficult for a single vaccine to effectively target all tumor cell variants.

Some efforts can be made to overcome these limitations in the future. Developing personalized vaccines based on individual tumor characteristics is expected to gain prominence. This would involve identifying unique tumor antigens and developing vaccines that specifically target these antigens, maximizing the immune response and therapeutic efficacy. The development of more efficient and targeted delivery systems for vaccines is also crucial. Nanotechnology-based approaches, for instance, could enhance the stability, bioavailability, and targeted delivery of vaccine components to immune cells, thereby improving the immune response. Also, selecting optimal adjuvants is another way to improve the vaccine's efficacy. In addition, multivalent vaccines containing multiple tumor-associated antigens, rather than targeting a single antigen, were designed and developed. This will amplify the immune response and reduce the risk of tumor escape. Moreover, combining NSCLC vaccines with other existing treatment modalities, such as immune checkpoint inhibitors, chemotherapy, radiotherapy, or targeted therapies, is likely to be a major focus. Synergistic effects of these combinations could enhance the overall anti-tumor immunity and improve patient outcomes. Overall, the future of NSCLC vaccines holds great promise, but it requires continued research and innovation in multiple areas to translate these potential benefits into improved patient survival and quality of life.

## Conclusions

This review provides an overview of the advancements in clinical trials for vaccine development in NSCLC. The number of vaccines clinical trials for NSCLC has continued to increase in recent years, with peptide/protein vaccines accounting for the largest proportion. Moreover, other types of vaccines like tumor cell vaccines, DNA/RNA vaccines, viral vector vaccines, and DC vaccines have also received considerable attention and are under thorough investigation. Despite the great potential and bright future of vaccines, most vaccines in NSCLC are still in the initial phases of research. Substantial efforts have been dedicated to surmounting the existing obstacles. To advance the field further, future research should focus on enhancing the immunogenicity and specificity of vaccines. This could involve optimizing the design of vaccine components, improving delivery methods, and combining vaccines with other therapeutic modalities such as immunotherapy or chemotherapy. These initiatives are critical to the development of more effective and personalized vaccines for NSCLC.

## Data Availability

Data sharing is not applicable to this article as no new data were created or analyzed in this study.
